# Supplementary material for: Comparison of the Efficacy of Empagliflozin, Dapagliflozin, and Allopurinol Based on Serum Uric Acid Levels and Kidney Function in Patients with Type 2 Diabetes Mellitus: A Retrospective Cohort Study
Source: Med Sci (Basel). 2025 Dec 26;14(1):12. doi: 10.3390/medsci14010012 (PMC12821499; doi:10.3390/medsci14010012)
Supplement: Supplementary file 1 [file medsci-14-00012-s001.zip › medsci-4037299-supplementary.pdf]

SUPPLEMENTARY FILE

Comparison of the Efficacy of Empagliflozin, Dapagliflozin and Allopurinol Based on Serum Uric Acid Levels and Kidney Function in Patients with Type 2 Diabetes Mellitus: A Retrospective Cohort Study

Roland Fejes, Tamás Jámbor, Tamás Lantos and Szabolcs Péter Tallósy

Supplementary Figure S1. Changes in chronic kidney disease (CKD) stage, mortality, and therapy discontinuation during the 36-month follow-up, visualized by alluvial plots. Each alluvial plot represents patient transitions between CKD stages or outcomes (death, therapy discontinuation) over time. Panels (a), (b), and (c) correspond to patients treated with dapagliflozin, empagliflozin, and allopurinol, respectively. The width of each flow is proportional to the number of patients moving between categories. Color-coding reflects CKD stage at each time point. CKD stages were determined according to the eGFR-based staging system described in the KDIGO guidelines. **G1:** eGFR ≥ 90, **G2:** eGFR 60–89, **G3a:** eGFR 45–59, **G3b:** eGFR 30–44, **G4:** eGFR 15–29

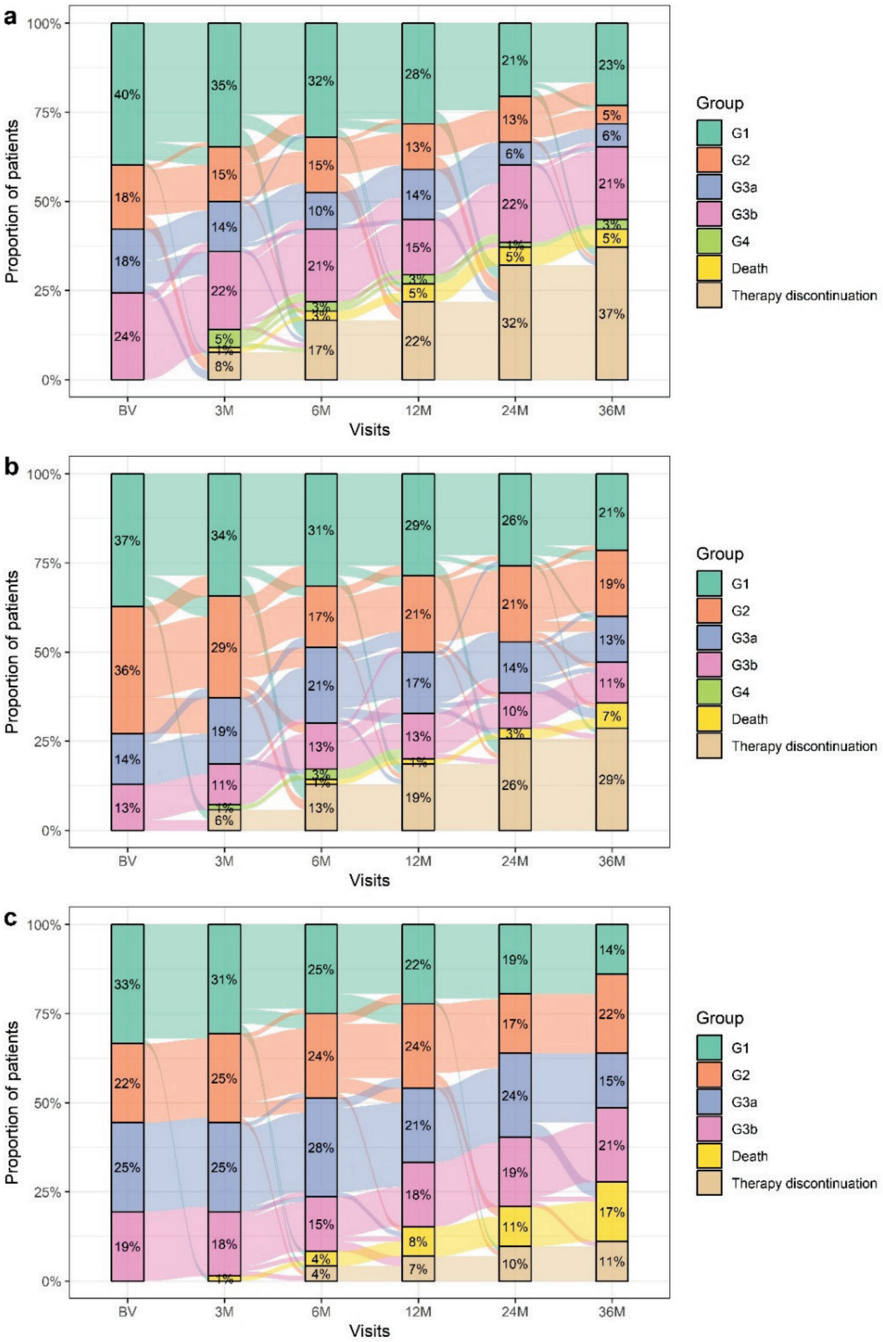

Supplementary Material S1 Detailed statistical results related to the longitudinal analysis of serum uric acid (sUA) changes.

The analysis was conducted using a mixed-effects model, which accounts for both fixed and random sources of variation. The dataset was organized in a stacked structure appropriate for repeated-measures designs. Sphericity was not assumed, indicating that variances and covariances across time points were allowed to differ. The significance level (α) was set at 0.05.

The Type III fixed effects analysis revealed that time exerted a statistically significant influence on the dependent variable ($F(1.419, 234.7) = 21.30$, $P < 0.0001$). Similarly, the treatment type had a significant effect ($F(2, 211) = 5.183$, $P = 0.0063$). However, the interaction between time and treatment type was not significant ($F(10, 827) = 0.9360$, $P = 0.4990$).

With regard to random effects, variability attributable to individual differences between subjects was moderate, with a standard deviation (SD) of 51.73 and a corresponding variance of 2676. The residual variability, representing unexplained variation, was larger (SD = 99.70, variance = 9940), suggesting that inter-individual differences accounted for a smaller proportion of the total variance compared with within-subject or random error variability.

The matching effectiveness test confirmed the appropriateness of the repeated-measures structure. The chi-square statistic was 79.46 with 1 degree of freedom, yielding a highly significant P value (< 0.0001). Consequently, the model's treatment of within-subject correlations can be considered valid and the estimated effects reliable.

Detailed sUA concentrations and corresponding sample sizes, stratified by treatment group and time point, are presented as follows:

**Serum uric acid (µmol/L)**			
	**Allopurinol**	**Dapagliflozin**	**Empagliflozin**
**BV**	430 (387; 476)	450 (419; 500)	452 (401; 496)
**3-month**	355 (309; 389)	396 (345; 436)	401 (370; 455)
**6-month**	340 (297; 391)	384 (312; 416)	398 (345; 430)
**12-month**	340 (302; 397)	361 (323; 410)	395 (354; 426)
**24-month**	327 (301; 376)	359 (313; 428)	387 (345; 417)
**36-month**	345 (320; 389)	364 (328; 399)	399 (362; 431)

The detailed results of Dunnett's multiple comparisons test (longitudinal comparisons within each treatment group) are presented below:

	Mean Difference	P Value
**Dapagliflozin**		
**BV vs. 3-month**	67.57	<0.0001
**BV vs. 6-month**	44.28	0.7925
**BV vs. 12-month**	92.28	<0.0001
**BV vs. 24-month**	90.39	<0.0001
**BV vs. 36-month**	90.67	<0.0001
**Empagliflozin**		
**BV vs. 3-month**	47.12	<0.0001
**BV vs. 6-month**	62.76	<0.0001
**BV vs. 12-month**	69.65	<0.0001
**BV vs. 24-month**	79.88	<0.0001
**BV vs. 36-month**	81.58	<0.0001
**Allopurinol**		
**BV vs. 3-month**	83.07	<0.0001
**BV vs. 6-month**	93.80	<0.0001
**BV vs. 12-month**	89.68	<0.0001
**BV vs. 24-month**	96.47	<0.0001
**BV vs. 36-month**	87.01	<0.0001

The detailed results of Tukey's multiple comparisons test (inter-group comparisons within time points) are presented below

	Mean Difference	P Value
**BV**		
**Dapagliflozin vs. Empagliflozin**	2.16	0.9788
**Dapagliflozin vs. Allopurinol**	19.78	0.2061
**Empagliflozin vs. Allopurinol**	17.62	0.3311
**3-month**		
**Dapagliflozin vs. Empagliflozin**	-18.29	0.3369
**Dapagliflozin vs. Allopurinol**	35.27	0.0211
**Empagliflozin vs. Allopurinol**	53.57	<0.0001
**6-month**		
**Dapagliflozin vs. Empagliflozin**	20.64	0.8995
**Dapagliflozin vs. Allopurinol**	69.29	0.3118
**Empagliflozin vs. Allopurinol**	48.65	<0.0001
**12-month**		
**Dapagliflozin vs. Empagliflozin**	-20.47	0.276
**Dapagliflozin vs. Allopurinol**	17.18	0.4421
**Empagliflozin vs. Allopurinol**	37.64	0.0096
**24-month**		
**Dapagliflozin vs. Empagliflozin**	-8.356	0.8721
**Dapagliflozin vs. Allopurinol**	25.86	0.207
**Empagliflozin vs. Allopurinol**	34.21	0.0427
**36-month**		
**Dapagliflozin vs. Empagliflozin**	-6.928	0.8925
**Dapagliflozin vs. Allopurinol**	16.12	0.5285
**Empagliflozin vs. Allopurinol**	23.05	0.1333

Supplementary Material S2 Detailed statistical results related to the longitudinal analysis of estimated glomerular filtration rate (eGFR) changes.

The analysis was conducted using a mixed-effects model, which accounts for both fixed and random sources of variation. The dataset was organized in a stacked structure appropriate for repeated-measures designs. Sphericity was not assumed, indicating that variances and covariances across time points were allowed to differ. The significance level (α) was set at 0.05.

The Type III fixed effects analysis demonstrated significant effects of time, treatment type, and their interaction. The main effect of time was statistically significant ($F(3.914, 647.4) = 5.549$, $P = 0.0002$). The treatment type also showed a significant effect ($F(2, 211) = 3.793$, $P = 0.0241$). Moreover, a strong and statistically significant time × treatment type interaction was observed ($F(10, 827) = 20.95$, $P < 0.0001$).

Regarding random effects, inter-individual variability was moderate, with a subject-level SD of 19.93 and a variance of 397.3. The residual variability, reflecting within-subject or unexplained variance, was lower (SD = 5.045, variance = 25.45).

The matching effectiveness test yielded a highly significant result ($\chi^2 = 1995$, df = 1, $P < 0.0001$), confirming that subject-level matching was successful and that the repeated-measures structure was statistically appropriate.

Detailed eGFR concentrations and corresponding sample sizes, stratified by treatment group and time point, are presented as follows:

**Estimated glomerular filtration rate (mL/min/1.73 m^2)**			
	**Allopurinol**	**Dapagliflozin**	**Empagliflozin**
**BV**	66 (52; 90)	68 (45; 90)	77 (58; 90)
**3-month**	68 (49; 89)	64 (40; 90)	72 (57; 90)
**6-month**	67 (49; 84)	69 (41; 90)	66 (57; 90)
**12-month**	63 (47; 80)	66 (45; 90)	73 (58; 90)
**24-month**	59 (44; 79)	78 (39; 90)	75 (58; 90)
**36-month**	58 (45; 74)	82 (37; 90)	73 (58; 90)

The detailed results of Dunnett's multiple comparisons test (longitudinal comparisons within each treatment group) are presented below:

	Mean Difference	P Value
**Dapagliflozin**		
**BV vs. 3-month**	2.77	<0.0001
**BV vs. 6-month**	0.7338	0.7104
**BV vs. 12-month**	1.788	0.1002
**BV vs. 24-month**	1.378	0.4431
**BV vs. 36-month**	0.1488	0.9997
**Empagliflozin**		
**BV vs. 3-month**	2.373	0.1125
**BV vs. 6-month**	3.749	0.0128
**BV vs. 12-month**	1.556	0.3432
**BV vs. 24-month**	0.3086	0.9997
**BV vs. 36-month**	1.882	0.555
**Allopurinol**		
**BV vs. 3-month**	1.049	0.0234
**BV vs. 6-month**	3.231	0.0001
**BV vs. 12-month**	4.665	<0.0001
**BV vs. 24-month**	8.222	<0.0001
**BV vs. 36-month**	9.705	<0.0001

The detailed results of Tukey's multiple comparisons test (inter-group comparisons within time points) are presented below

	Mean Difference	P Value
**BV**		
**Dapagliflozin vs. Empagliflozin**	-6.897	0.1099
**Dapagliflozin vs. Allopurinol**	-1.737	0.8776
**Empagliflozin vs. Allopurinol**	5.161	0.2534
**3-mont.**		
**Dapagliflozin vs. Empagliflozin**	-7.294	0.1221
**Dapagliflozin vs. Allopurinol**	-3.457	0.6336
**Empagliflozin vs. Allopurinol**	3.837	0.4913
**6-month**		
**Dapagliflozin vs. Empagliflozin**	-3.883	0.5779
**Dapagliflozin vs. Allopurinol**	0.7601	0.978
**Empagliflozin vs. Allopurinol**	4.643	0.3684
**12-month**		
**Dapagliflozin vs. Empagliflozin**	-7.13	0.17
**Dapagliflozin vs. Allopurinol**	1.14	0.9542
**Empagliflozin vs. Allopurinol**	8.27	0.0437
**24-month**		
**Dapagliflozin vs. Empagliflozin**	-7.967	0.1344
**Dapagliflozin vs. Allopurinol**	5.107	0.4275
**Empagliflozin vs. Allopurinol**	13.07	0.0009
**36-month**		
**Dapagliflozin vs. Empagliflozin**	-5.164	0.4866
**Dapagliflozin vs. Allopurinol**	7.819	0.1838
**Empagliflozin vs. Allopurinol**	12.98	0.0025
